# Building behavior does not drive rates of phenotypic evolution in spiders

**DOI:** 10.1073/pnas.2102693118

**Published:** 2021-08-09

**Authors:** Jonas O. Wolff, Kaja Wierucka, Gabriele Uhl, Marie E. Herberstein

**Affiliations:** ^a^Department of Biological Sciences, Macquarie University, Sydney, NSW 2109, Australia;; ^b^Zoological Institute and Museum, University of Greifswald, 17489 Greifswald, Germany;; ^c^Department of Anthropology, University of Zurich, 8057 Zurich, Switzerland

**Keywords:** extended phenotype, animal architecture, niche construction, Araneae

## Abstract

Do animals set the course for the evolution of their lineage when manipulating their environment? This heavily disputed question is empirically unexplored but critical to interpret phenotypic diversity. Here, we tested whether the macroevolutionary rates of body morphology correlate with the use of built artifacts in a megadiverse clade comprising builders and nonbuilders—spiders. By separating the inferred building-dependent rates from background effects, we found that variation in the evolution of morphology is poorly explained by artifact use. Thus natural selection acting directly on body morphology rather than indirectly via construction behavior is the dominant driver of phenotypic diversity.

The idea that organisms actively change their environment and thereby influence natural selection acting upon them is considered a major challenge to traditional evolutionary theory (1). This is especially apparent in artifacts constructed by animals (e.g., nests, burrows, capture webs) that blur the boundaries between an organism’s phenotype and its environment. Such artifacts have been interpreted as a part of the adaptive repertoire of an organism and directly controlled by its genes—so-called “extended phenotypes” (2). In addition, animal artifacts could act as ecological factors that modulate natural selection acting on the constructor, an idea most prominently brought about by the “niche construction perspective” (3–5). For instance, the repeated exposure to the genetically determined environment across generations may result in the homogenization of selection pressures because the constructed environment is less variable than the autonomous environment and, as a result, the deceleration of somatic evolution (6). Alternatively, the correlated evolution of building behavior and builder morphology could lead to an acceleration of somatic evolution after a novelty in construction behavior arises (such as gain or loss of web building) (6).

The core of both extended phenotype and niche construction perspectives is that the behavioral interaction with the environment poses a significant bias to the evolution of physical traits, an old idea that has recently gained increasing popularity (7). The proposal that artifact construction biases evolution has been heavily disputed (1, 8, 9). This hypothesis of construction behavior leading to a distinguished evolutionary dynamic (6), however, has mainly been explored theoretically (e.g., ref. 5) and never tested on a larger scale with actual traits and phylogenetic data. Clarifying the role of construction behavior in macroevolution is crucial as it is one of the main arguments for the claim that evolutionary theory needs to be extended (10).

Here, we put this hypothesis to the test by exploring the effect of construction behavior on morphological evolution, while controlling for background effects. To this aim, we used a megadiverse (47,000 species) and ubiquitous order of animals with the largest variation of built structures observable—spiders (Araneae) (11, 12). Spiders are a prime model to study effects of artifact construction on organismal evolution. As some lineages spend almost all of their time in webs while others do not build webs, they provide a unique opportunity to directly compare the evolutionary pathways of closely related builders and nonbuilders. As multiple convergent events of web loss and regain are distributed across the spider tree of life (13), there are sufficient replicates to attempt to draw a general conclusion. While all spiders are able to produce silk, the extend of construction behavior differs enormously between builders and nonbuilders: builders construct and dwell in webs or silk-lined burrows, that are involved in foraging and provide a protective and “predictable” environment; nonbuilders, on the other hand, interact directly with variable habitats and use their body for prey capture. Spiders of both guilds usually wrap their eggs in silk and, depending on their lifestyle, suspend their cocoons in the web or attach them to microhabitat surfaces. Spiders thus not only interact with the environment itself, affected by their genetically inherited behavior, but also with the environment constructed or chosen by their mother, indicating that the gene–environment relationship is nontrivial.

Utilizing the novel MuSSCRat (multiple state-specific rates of continuous-character evolution) approach (14), we compared the support (using Bayes factors) of two alternative evolutionary models to explain the observed variation of body sizes and shapes in a de novo compiled database of a representative sample of the extant spider fauna. The first model (state-dependent model) assumed that the rates of phenotypic evolution differ between lineages that spend most of their lifetime in a constructed environment (i.e., web or burrow, state 1) and lineages that forage and reproduce in a nonconstructed environment (state 0), while the alternative model (state-independent model) excluded such a difference. Both models assumed that there are alternative sources of variation (background effects) and separately inferred the state-dependent and background rates for each branch of the phylogenetic tree. This avoids an erroneous attribution of any found rate difference to the studied effect, known as the “straw-man” problem (14).

The following rationale was used, with ζ being the state-dependent evolutionary rate:

H_0_: State-independent model better explains observed trait variation; ζ[builder] = ζ[nonbuilder]. This would indicate that construction behavior does not significantly alter the dynamics of morphological evolution.
H_1_: State-dependent model better explains observed trait variation; ζ[builder] > ζ[nonbuilder]. This would indicate that construction behavior enhances the morphological diversification process.
H_2_: State-dependent model better explains observed trait variation; ζ[builder] < ζ[nonbuilder]. This would indicate that construction behavior reduces the morphological diversification process by stabilizing the niche space and buffering external selective pressures.

Both H_1_ and H_2_ have been proposed as scenarios consistent with the niche construction perspective (6).

In total, we ran two reversible-jump Markov chain Monte Carlo analyses over 0.5 million generations for each three datasets: (a) a body-size-only dataset with 815 species (with 26 gains and 46 losses of building behavior), (b) a body size + body shape dataset with 749 species (with 22 gains and 41 losses of building behavior), and (c) a dataset containing a combination of six general traits for 340 species (with 14 gains and 29 losses of building behavior) (*SI Appendix*, *Extended Methods*). We did not find significant evidence for H_1_ or H_2_ for the body size dataset [Bayes factor *BF*(H_0_) = 7.56; *BF*(H_1_ + H_2_) = 0.13; *BF*(H_1_) = 0.10; *BF*(H_2_) = 0.28; Fig. 1*A*] and the body size + body shape dataset [*BF*(H_0_) = 2.45; *BF*(H_1_ + H_2_) = 0.41; *BF*(H_1_) = 0.03; *BF*(H_2_) = 1.17; Fig. 1*B*]. For the six-traits dataset, there was a weak support for H_2_ [*BF*(H_0_) = 0.65; *BF*(H_1_ + H_2_) = 1.54; *BF*(H_1_) = 0.02; *BF*(H_2_) = 4.49; Fig. 1*C*]. However, comparison of the branch-specific evolutionary rates revealed that less than 15% of the rate variation was explained with state-dependent effects, most of which was driven by a single clade of nonbuilders, the Dionycha (Fig. 1 *C*, *Lower*). The results were robust toward different prior assumptions in the model (details in *SI Appendix*). The absence of a clear correlation between building behavior and rates of morphological evolution such as predicted by the niche construction perspective (H_1_ and H_2_) enables us to conclude that the proposed mechanism is most likely not significant in spider gross morphology.

**Fig. 1.**
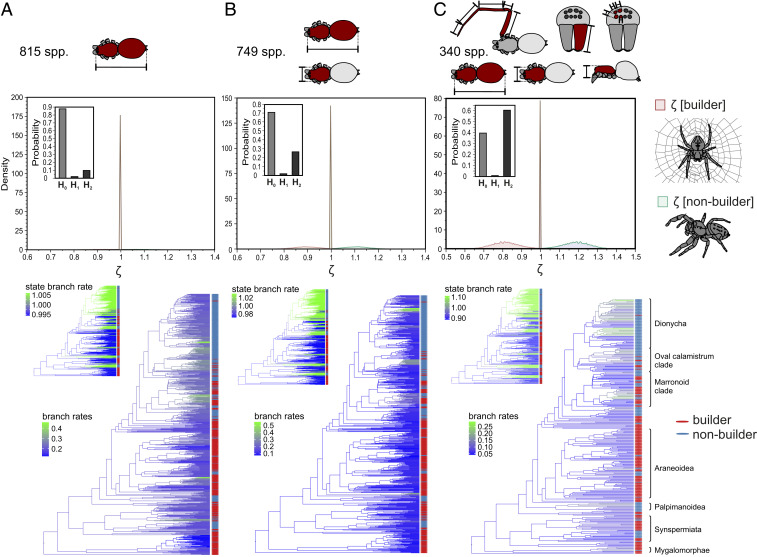
Posterior probability of niche construction effects on the rate of morphological evolution in spiders. Marginal posterior distribution of state-dependent rates (ζ) and posterior probability for each hypothesis (*Inset* bar plot, with H_0_ ζ[builder] = ζ[nonbuilder]; H_1_ ζ[builder] > ζ[nonbuilder]; H_2_ ζ[builder] < ζ[nonbuilder]), from two combined reversible-jump Markov chain Monte Carlo analyses with 0.5 million generations each. Below mapping of the average branch-specific and state-dependent (smaller *Inset*) evolutionary rates from the posterior distribution. Tip labels indicate the state of the behavioral character (red, builder; light blue, nonbuilder). (*A*) Body size evolution, 815 species. (*B*) Evolution of body size and shape, 749 species. (*C*) Evolution of body size, body shape, cephalothorax height, mouth part size, eye size, and leg length; 340 species. For a definition of traits, see *SI Appendix*, *Extended Methods*.

Per branch mapping of state-dependent, background, and global rates revealed that the rate variation was dominated by patterns that did not match the ecological states (Fig. 1, *Bottom*). Low and high rates occurred across both builders and nonbuilders. Exceptionally high rates were found in clades that exhibit mimicry (e.g., the myrmecomorphic Castianeirinae), masquerade (some orb web building Tetragnathidae), specialize on hazardous prey (e.g., the araneophagous Archaeidae and Gnaphosoidea), are associated with sheltering in crevices (e.g., the “flattie spiders” Selenopidae, Trochanteriidae, and Sparassidae), or radical habitat shifts (e.g., from forest to marine habitats in Desidae and Toxopidae). This indicates that predator–prey interactions and ecosystem changes are the dominant drivers of the phenotypic diversity in spiders.

We conclude that, at least in the case of spiders, artifact use does not significantly modify the rates of evolutionary change in body size and shape. This suggests that the construction of shelters and webs does not necessarily buffer the environmental selective pressures acting upon animal body shapes, nor that its evolution accelerates phenotypic diversification processes. Rather, construction behavior is one of the many responses to selective pressures similar to somatic adaptations. Our results do not support the increasingly popular idea that behavior universally acts as a pacemaker in evolution and plays an important role in the rapid adaptation to a changing world (6, 7, 15).

## Supplementary Material

Supplementary File

Supplementary File

Supplementary File

Supplementary File

Supplementary File

## Data Availability

Raw data and scripts have been deposited in Dryad (DOI: 10.5061/dryad.tb2rbp015). All other study data are included in the article and/or supporting information.
