# Supplementary material for: Building behavior does not drive rates of phenotypic evolution in spiders
Source: Proc Natl Acad Sci U S A. 2021 Aug 9;118(33):e2102693118. doi: 10.1073/pnas.2102693118 (PMC8379907; doi:10.1073/pnas.2102693118)
Supplement: Supplementary File [file pnas.2102693118.sapp.pdf]

Building behaviour does not drive rates of phenotypic evolution in spiders

Jonas O. Wolff, Kaja Wierucka, Gabriele Uhl, Marie E. Herberstein

SI Appendix. Extended Methods

Trait database

We built a database of morphometric and ecological data on a representative taxon sample of the order Araneae. We followed the taxon sample of the Araneae Tree of Life project (AToL) [1], which contains 932 terminals of all at that time valid families except Synaphridae (corresponds to ~2% of described species). This sample is representative of the phylogenetic and morphological diversity of spiders. AToL terminals that were not identified to species level and for which no image material was available were replaced with described species with a type locality close to the collection site (26.3% of the used sample; details in S2). 11% of the AToL terminals were omitted as there was not enough information to determine a suitable replacement, resulting in a total of 828 included species.

The morphological data were assembled by extracting data from taxonomic descriptions using the WSC database [2], and measurements on images published in articles or online repositories (including Morphbank :: Biological Imaging, http://www.morphbank.net, where images of AToL specimens were deposited), with one to seven sources combined per species (for statistics of used sources see S2, and for a list see S3). As many spiders exhibit a significant sexual dimorphism, only data of adult females were used. We included only general traits, i.e., ones that were assumed to be affected by more than one niche property. For instance, body shape may be under selection from a mix of abiotic (e.g., temperature and microhabitat structure) and biotic (e.g., prey spectrum and predation) factors. The following measurements were recorded: body length; cephalothorax (prosoma) length; cephalothorax width; height of cephalothorax (carapace); length of mouth parts (i.e. cheliceral base segment); diameter of each eye type; length of front leg (excl. coxa, trochanter and pretarsus). From these the following six traits were calculated: (1) body size (=body length); (2) body shape (cephalothorax width / cephalothorax length); (3) relative cephalothorax height (cephalothorax height / (cephalothorax length + width)); (4) size of mouth parts (paturon length / cephalothorax height); (5) eye size

(sum of diameters of all eye types / cephalothorax width); (6) relative leg length (length of front leg / cephalothorax width). From each trait the species mean was calculated (i.e., from the 1-7 data sources, for details see S2 and S3) and log-transformed, to build the species matrix for further analysis (S5).

The ecological data matrix was built by assessing the literature on same or closely related species, and in few cases complemented by personal observations (for details, see S4). We used a binary coded category: state 0, non-builder; state 1, builder. We defined a species as a 'builder' (1), if individuals spend most of their life in a self-constructed web or burrow, i.e. foraging and reproduction takes place on, in or from the artefact, and the artefact aids in prey capture, signalling and/or defence. In contrast, a 'non-builder' (0) does not build a capture web or a burrow, it may build a retreat, which, however, is only used in periods of inactivity and does not aid in prey capture.

Test of evolutionary hypothesis

To infer state-dependent evolutionary rates the recent MuSSCRat (multiple state-specific rates of continuous-character evolution) approach was used [3]. MuSSCRat is a reversible-jump Markov chain Monte Carlo (rjMCMC) approach to determine the likelihood of a model where a discrete (ecological) trait correlates with the evolutionary rates of one or more continuous traits vs. a model where no such relationship exists. In both models, it is assumed that there are alternative (background) effects on rate variation, which is implemented by partitioning the inferred global rates into a state-dependent and a background rate domain (for details, see Burress et al. [4]). This avoids the pitfall of traditional approaches where any difference in the evolutionary mode between two ecological groups is attributed to the trait state (known as the 'straw-man argument' problem [3]). The analysis was built in *RevBayes* [5], based on the code by Burress et al. [4], with slight modifications (scripts and input files accessible from Dryad repository, doi:10.5061/dryad.tb2rbp015). As the phylogenetic model we used a time-calibrated ultrametric tree including all AToL terminals [6]. The evolution of the discrete character followed a Mk model [7], with the rate parameter λ drawn from a log-uniform distribution (a=0.0001, b=1). For the two multivariate datasets a LKJ prior with eta $\eta = 1.0$ and dim = n (characters) was used for the partial correlation matrix. For the background-rates model the assumption that characters evolve at a constant rate was relaxed by applying a relaxed local clock prior, as described in Burress et al. [4]. The prior for the expected number of rate changes was set equivalent to the number of transitions between the states of the discrete trait. This

number was inferred *a priori* by ancestral character estimation in *phytools* using ER and ARD models with the stochastic character mapping approach with 100 iterations [8]. To effectively sample between both the state-dependent and the state-independent model a weight of ten was set on the reversible jump proposal. Each of two chains were run over 500,000 generations for (a) a body size only dataset with 815 species, (b) a body size + body shape (as defined under 2.1 above) dataset with 749 species, and (c) a dataset containing all six traits (as defined under 2.1 above) for 340 species. Parameters were sampled every 10th generation. The combined log files of both chains were analyzed in *Tracer* 1.7.1 [9] to assess convergence and summarize parameters, with the exclusion of a burn-in of 10%. The posterior probability of H1 was computed as the fraction of MCMC samples for which $\zeta(1) > \zeta(0)$. The posterior probability of H2 was computed as the fraction of MCMC samples for which $\zeta(1) < \zeta(0)$. Bayes factors (BF) were calculated by dividing the posterior odds (the ratio of posterior probabilities of the competing models) by the corresponding prior odds. BFs were interpreted, using the guidelines in Kass and Raftery (i.e., evidence against competing hypotheses BF > 3; strong evidence if BF > 20) [10]. Branch-specific rates were visualized in *RevGadgets* (https://github.com/revbayes/RevGadgets).

Sensitivity analysis

To test the sensitivity of the analysis towards the prior estimated number of rate shifts, we ran additional tests using an expected number of shifts of 0.1 and 0.01 times the number of branches with chains of 100,000 generations each. The effects were similar as shown in Burress et al. [4], i.e. the state dependent evolutionary rates ζ, the posterior evolutionary rate at the root β^2_R, the rate of building behaviour λ and the number of state changes was consistent across different priors. There was an effect on the posterior number of rate changes, but this did not affect the locations of major rate shifts. The posterior probability of the different hypotheses H_0, H_1 and H_2 was slightly affected by the prior on the expected number of rate shifts: H_2 tended to have a stronger support for a very low prior number of rate shifts. In the case of the body length only and the 6-traits datasets, this had no effect on the significance category after Kass and Raftery [10]. In the case of the 2-traits dataset, there was weak support for H_2 ($BF(H_0) = 0.44$; $BF(H_2) = 6.70$) at a prior of 0.01 times the number of branches, while for a prior of 0.1 times the number of branches there was weak support for H_0 ($BF(H_0) = 5.35$; $BF(H_2) = 0.48$). Notably, in all cases, there were only slight effects on the magnitude of state-dependent rates, the locations of major rate shifts were consistent throughout priors, and only a minor fraction

of the rate variation was explained with state-dependent effects. We therefore conclude that our reported results are robust.

Visual representations of the results from the sensitivity analyses can be accessed from the Dryad repository (doi:10.5061/dryad.tb2rbp015).

Effect of data sample on the results

We performed three separate analyses based on the three datasets differing in the number of species and traits. The results showed some effects of the data sample on the posterior probability of hypotheses H_0 and H_2 (as reported in main text and in the section above). Using the 6-traits dataset led to a higher support of H_2 than using the either of the other two datasets that included more than double as many species but less traits. To test if the taxon sample of the 6-traits dataset is biased towards H_2 we ran two additional chains over 100,000 generations with this dataset, but only including body length. Results barely differed from the results obtained with the larger body length-only dataset (analysis of body length evolution with taxon sample of 6-traits dataset: $BF(H_0) = 6.50$; $BF(H_1+H_2) = 0.15$; $BF(H_1) = 0.32$; $BF(H_2) = 0.11$; with taxon sample of body length-only dataset: $BF(H_0) = 7.56$; $BF(H_1+H_2) = 0.13$; $BF(H_1) = 0.10$; $BF(H_2) = 0.28$), indicating that the reduced taxon sample of the 6-traits dataset is not biased towards H_2. This suggests that the observed differences in hypothesis support between datasets are explained by the different number of included traits rather than the different number of included taxa. We thus cannot rule out that H_2 might become more likely for some specific combinations of traits not tested here. However, as for none of the tested cases (i.e., increasing either taxon sampling or trait number) there was a strong support for H_1 or H_2, we are confident that our conclusions are robust and can be generalized. We note the 6-traits dataset includes descriptors of all major body parts (cephalothorax, abdomen, legs, mouth parts, and eyes) and thus well describes spider gross morphology. We argue that if niche construction has a significant effect on phenotypic evolution, it should leave its trace in such general descriptors and not in very specific traits directly associated with the building behaviour (such as the size of silk glands). Comparison of the branch-specific, state-dependent, and global rates showed that if H_2 is accepted, it only explains a very small fraction of rate variation. Rate shifts and changes (losses or gains) in building behaviour were almost uncorrelated. This was consistent across datasets.

Method References

1 Wheeler, W. C. *et al.* The spider tree of life: phylogeny of Araneae based on target-gene analyses from an extensive taxon sampling. *Cladistics* **33**, 574-616 (2017).

2 Nentwig, W., Gloor, D. & Kropf, C. Taxonomic database: Spider taxonomists catch data on web. *Nat Cell Biol* **528**, 479 (2015).

3 May, M. R. & Moore, B. R. A Bayesian Approach for Inferring the Impact of a Discrete Character on Rates of Continuous-Character Evolution in the Presence of Background-Rate Variation. *Syst Biol* **69**, 530-544 (2020).

4 Burress, E. D., Martinez, C. M. & Wainwright, P. C. Decoupled jaws promote trophic diversity in cichlid fishes. *Evolution* **74**, 950-961 (2020).

5 Höhna, S. *et al.* RevBayes: Bayesian phylogenetic inference using graphical models and an interactive model-specification language. *Syst Biol* **65**, 726-736 (2016).

6 Fernández, R. *et al.* Phylogenomics, diversification dynamics, and comparative transcriptomics across the spider tree of life. *Curr Biol* **28**, 1489-1497 (2018).

7 Lewis, P. O. A likelihood approach to estimating phylogeny from discrete morphological character data. *Syst Biol* **50**, 913-925 (2001).

8 Revell, L. J. phytools: an R package for phylogenetic comparative biology (and other things). *Methods Ecol Evol* **3**, 217-223 (2012).

9 Rambaut, A., Drummond, A. J., Xie, D., Baele, G. & Suchard, M. A. Posterior summarization in Bayesian phylogenetics using Tracer 1.7. *Syst Biol* **67**, 901 (2018).

10 Kass, R. E. & Raftery, A. E. Bayes factors. *J Am Stat Assoc* **90**, 773-795 (1995).
